# Microbiome-based therapeutics for *Clostridioides difficile* infection: additive, subtractive, and modulatory strategies in modern clinical practice

**DOI:** 10.1080/19490976.2026.2722482

**Published:** 2026-08-27

**Authors:** Roxana-Elena Cristian, Corneliu Ovidiu Vrancianu, Marian Constantin, Mihaela Paun, Eduard C. Milea, Dragos Malaescu, Mariana Carmen Chifiriuc

**Affiliations:** a National Institute of Research and Development for Biological Sciences, Bucharest, Romania; b The Research Institute of the University of Bucharest (ICUB), Bucharest, Romania; c Doctoral School, Carol Davila University of Medicine and Pharmacy, Bucharest, Romania; d Department of Microbiology, Institute of Biology of the Romanian Academy, Bucharest, Romania; e Faculty of Administration and Business, University of Bucharest, Bucharest, Romania; f Public Health Directorate, Bucharest, Romania; g Microbiology-Immunology Department, Faculty of Biology, University of Bucharest, Bucharest, Romania; h Biological Sciences Division, Romanian Academy, Bucharest, Romania

**Keywords:** Microbiome therapeutics, *Clostridioides difficile*, fecal microbiota transplantation, live biotherapeutic products, defined microbial consortia, anti-toxin therapy

## Abstract

Microbiome-based therapeutics have rapidly evolved into a transformative field at the intersection of infectious diseases, microbial ecology, and precision medicine. *Clostridioides difficile* infection (CDI) represents the most extensively studied model, providing a framework for understanding microbiome-driven disease and ecological restoration. This review synthesizes three major therapeutic strategies, additive, subtractive, and modulatory, and integrates recent advances from basic, translational, and clinical research. Additive approaches, including fecal microbiota transplantation (FMT), live biotherapeutic products (LBPs), and defined microbial consortia, aim to restore microbial diversity and colonization resistance. Subtractive strategies selectively target *C. difficile* or its ecological advantages through targeted antibiotics, bacteriocins, bacteriophages, and CRISPR-based antimicrobials. Modulatory therapies reshape host–microbe and microbe–microbe interactions, targeting toxin activity, bile acid metabolism, and spore germination. Together, these approaches reflect a paradigm shift from pathogen-centered treatment toward ecological therapeutics targeting the dysbiotic niche underlying CDI persistence and recurrence. Understanding the similarities and differences between these strategies can help guide the design of future microbiome-targeted interventions and explore their potential beyond CDI across microbiome-mediated diseases.

## Graphical abstract

**Figure:**
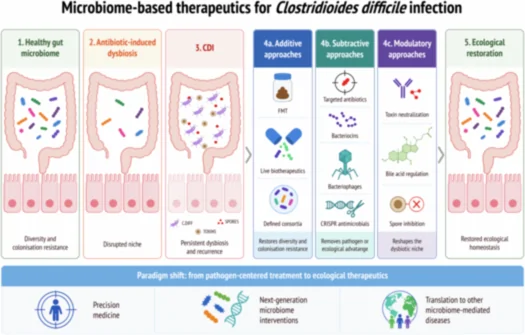

## 1. Introduction

The human microbiome represents a highly diverse ecosystem of bacteria, fungi, viruses, and archaea that colonize distinct physiological niches, particularly the gastrointestinal tract.^1‐3^ Far from functioning as a passive microbial backdrop, these communities actively participate in digestion, energy metabolism, immune maturation, and the maintenance of mucosal barrier integrity.^4‐7^ Advances in sequencing technologies and multi-omics approaches have substantially expanded our understanding of how microbiome composition and function contribute to both health and disease.^8^ ^,^ ^9^ Dysbiosis, defined as the disruption of microbial balance, has been implicated in a wide range of pathological conditions, from gastrointestinal disorders such as inflammatory bowel disease to systemic diseases including metabolic, cardiovascular, autoimmune, and neuropsychiatric conditions.^10‐14^ Beyond compositional changes, microbiome-derived metabolites and ecological interactions play a central role in shaping host physiology and disease susceptibility, highlighting the importance of functional, rather than purely taxonomic, characterization.^6^

In consequence, microbiome-derived signatures are increasingly explored as predictive biomarkers of treatment response and disease progression, supporting the emergence of precision microbiome-based medicine. The microbiome-centered therapeutics encompass a broad spectrum of approaches, ranging from the addition of beneficial microorganisms to the selective elimination of pathogens and the targeted modulation of host–microbiome interactions to restore ecological homeostasis. The main advantage of these approaches, unlike host-directed interventions, is that microbial communities can be reshaped through diverse and relatively accessible strategies, including dietary modification, probiotics, fecal microbiota transplantation (FMT), and more recently, bacteriophage-based therapies and engineered microbial platforms.^15‐21^

Among microbiome-associated diseases, *Clostridioides difficile* infection (CDI) represents the most clinically established model linking dysbiosis to disease pathogenesis and therapeutic response. CDI typically arises following antibiotic-induced disruption of the gut microbiota, leading to loss of colonization resistance and uncontrolled pathogen expansion. Importantly, CDI is also one of the few conditions in which restoration of the microbiome can achieve sustained clinical cure, making it an ideal framework for understanding microbiome-targeted interventions.

Despite its rapid expansion, the microbiome-based medicine field remains yet conceptually fragmented, the microbiome composition, metabolic function, host immune responses, and therapeutic strategies being predominantly investigated in isolation. Specifically, recent clinical studies highlight a disconnect between immunological or microbiological endpoints and clinical protection, underscoring the difficulty of establishing causal relationships and the complexity of translating mechanistic insights into predictive and clinically actionable frameworks as well as effective interventions. Moreover, interindividual variability in microbiome composition complicates reproducibility, while safety concerns and regulatory challenges persist, particularly for complex biological interventions such as FMT.^16^ ^,^ ^22^ ^,^ ^23^ These limitations remain important barriers to implementation in routine clinical practice.

Nevertheless, recent regulatory advances and increasingly standardized clinical trial frameworks suggest that the field is entering a phase of accelerated clinical maturation.^24^ ^,^ ^25^

In this context, our review is proposing a systems-level, integrative perspective on CDI, by integrating evidence from basic, translational, and clinical studies. Our triple aim is to (i) bridge currently disconnected research areas through the integration of multi-omics evidence in microbial, metabolic, immunological, and therapeutic fields within a coherent conceptual framework; (ii) propose a mechanistic model linking dysbiosis to CDI disease progression and therapeutic response; and (iii) identify critical knowledge gaps that hinder causal inference and translational application. By shifting from descriptive and compartmentalized approaches to mechanism-driven integration, we provide a conceptual and translational roadmap for the development of predictive models and next-generation targeted therapies, with implications extending beyond CDI to other dysbiosis-driven diseases.

By delineating how additive, subtractive, and modulatory strategies converge and diverge in CDI, the review highlights key opportunities and persistent challenges, particularly those related to safety, reproducibility, mechanistic understanding, and regulatory pathways. Unlike recent reviews that focus primarily on microbiota restoration strategies, including FMT optimization and implementation, live biotherapeutic products, or selected emerging therapeutics,^26‐36^ as well as reviews centered on virulence-modulating and engineered microbiome approaches,^37^ this review adopts a disease-centered and systems-level perspective using CDI as a prototypical dysbiosis-driven disorder. We integrate microbial ecology, colonization resistance, metabolic dysfunction, host responses, and therapeutic mechanisms into a unified framework linking disease pathogenesis to microbiome-directed interventions. Current and emerging therapies are organized according to their ecological function, additive, subtractive, and modulatory, rather than by product class alone, and emphasize functional ecosystem restoration over taxonomic recovery as the primary therapeutic objective. By connecting mechanistic insights across the continuum from dysbiosis to recovery, this review provides a translational framework for the rational development of next-generation microbiome therapeutics in CDI and potentially other microbiome-mediated diseases.

## 2. Literature search strategy

A structured literature search was conducted to identify relevant studies addressing microbiome-based therapeutic strategies for CDI, with particular emphasis on additive, subtractive, and modulatory approaches. Electronic databases, including PubMed, Scopus, and Web of Science, were systematically searched for articles published between 2016 and 2026 to capture recent advances in microbiome restoration, targeted antimicrobial strategies, passive immunotherapy, and vaccine development for CDI. Earlier seminal studies were additionally included when necessary to provide the historical and mechanistic foundations of established therapeutic concepts. The search strategy combined keywords and controlled vocabulary terms related to CDI and microbiome therapeutics, including “*Clostridioides difficile*”, “microbiome therapeutics”, “fecal microbiota transplantation”, “live biotherapeutic products”, “microbiota restoration”, “bacteriophages”, “endolysins”, “bacteriocins”, “CRISPR antimicrobials”, “engineered probiotics”, “monoclonal antibodies”, “bezlotoxumab”, “vaccines”, “colonization resistance”, “bile acids”, and “recurrent CDI”, using Boolean operators (“AND”, “OR”) to refine results. Study selection followed a structured screening process based on title, abstract, and full-text evaluation. Peer-reviewed original research articles, clinical trials, observational studies, translational investigations, and relevant preclinical studies published in English were considered. Reviews were also examined to identify additional primary literature. Studies lacking mechanistic, experimental, or clinical relevance to CDI therapeutics were excluded.

## 3. Microbiome-based therapeutic approaches

**Figure 1.**
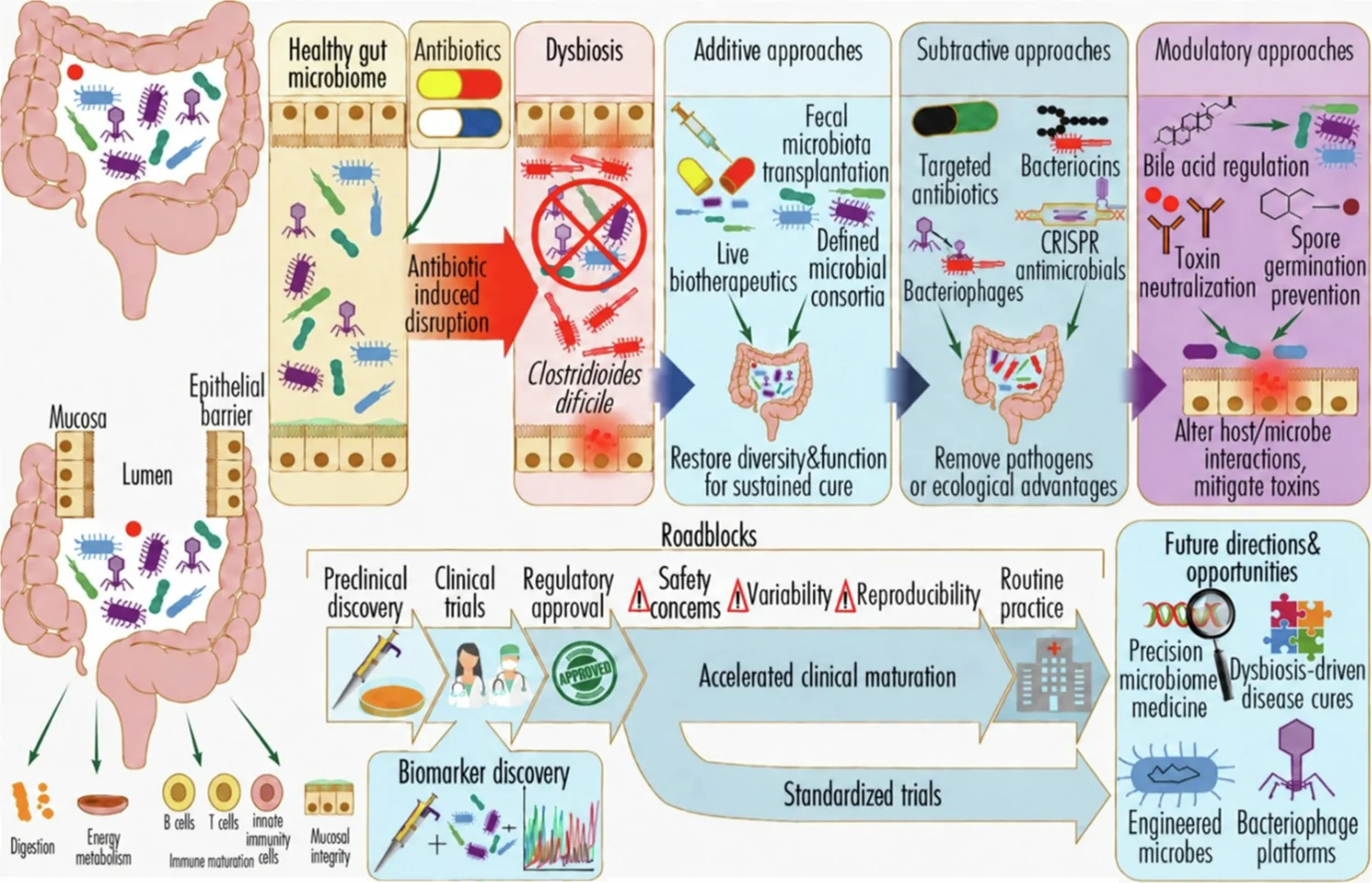
**Conceptual and mechanistic framework of microbiome-based therapeutics in CDI.** Antibiotic-induced dysbiosis disrupts colonization resistance, enabling *C. difficile* expansion and toxin-mediated epithelial damage. This state involves altered microbial composition, impaired metabolic function (notably bile acid metabolism), and disrupted host–microbiome interactions. Microbiome-based therapies can be grouped into three mechanistic strategies: additive approaches (e.g., fecal microbiota transplantation, live biotherapeutics, defined consortia) that restore microbial diversity and function; subtractive approaches (e.g., targeted antibiotics, bacteriophages, CRISPR-based antimicrobials) that selectively eliminate pathogens; and modulatory approaches that regulate host–microbe interactions, including toxin neutralization, bile acid metabolism, and spore germination. Key translational challenges include safety, interindividual variability, and reproducibility, while emerging directions point toward precision microbiome medicine and engineered microbial platforms (created with Adobe Illustrator CS6, Adobe Photoshop CS3).

Considerable efforts have been directed toward leveraging the advantages of host-microbiome interactions to advance microbiome-based therapeutics. Three commonly employed strategies in this endeavor include additive, subtractive, and modulatory therapy.^38^ Additive approaches aim to restore microbial diversity and function, subtractive strategies selectively eliminate *C. difficile* or its ecological advantages, while modulatory therapies act on host–microbe and microbe–microbe interactions, mitigating toxin activity, regulating bile acid metabolism, and preventing spore germination. Importantly, these categories should be viewed as a conceptual framework rather than a strict biological classification, as many therapeutic interventions exert overlapping effects across multiple domains. For example, microbiota-based therapies may simultaneously restore beneficial microbial communities, suppress pathogen expansion, and reshape microbial metabolism, whereas immune-based interventions can indirectly influence microbiome recovery and colonization resistance. These mechanistic strategies and their translational implications in CDI are summarized in Figure 1.

### 3.1. Additive therapy

Additive therapy refers to the introduction of beneficial microorganisms or microbial communities to restore or enhance the functions of the host microbiome. This category includes fecal microbiota transplantation (FMT),^38^ as well as next-generation approaches providing standardized and controlled alternatives to whole-microbiome transfer, such as live biotherapeutic products (LBPs)^39^ and defined microbial consortia.^40^ ^,^ ^41^ Together, these strategies aim to supplement or replace disrupted microbial populations to achieve therapeutic benefits.

#### 3.1.1. FMT therapy

Among microbiome-based interventions, FMT is the most established microbiota-restoration strategy for recurrent CDI (rCDI). Mechanistically, FMT restores colonization resistance through multiple complementary pathways, including re-establishment of microbial diversity, recovery of short-chain fatty acid production, and normalization of bile acid metabolism, all of which contribute to suppression of *C. difficile* germination and outgrowth. Microbiome profiling studies further support these ecological effects by demonstrating donor microbiota engraftment and sustained restructuring of microbial communities following transplantation. Persistent increases in microbial diversity and long-term alterations in microbial composition have been observed after FMT, supporting the concept that durable microbiome remodeling contributes to sustained clinical benefit.^42^ Despite these advances, the specific microbial taxa, metabolic pathways, and host–microbiome interactions responsible for therapeutic success remain incompletely understood.

The procedure involves transferring a complex microbial community from a healthy donor to a dysbiotic recipient, with the aim of re-establishing microbial diversity and ecological functions disrupted by antibiotic exposure.^43^ ^,^ ^44^ CDI represents a unique model for investigating donor–recipient microbial engraftment, as successful colonization by donor-derived microorganisms is increasingly recognized as a key determinant of therapeutic efficacy and long-term microbiome restoration.^45^ ^,^ ^46^ Early clinical and microbiome-based studies provided evidence that FMT could achieve sustained resolution of recurrent disease while restoring a disrupted intestinal microbial community. Khoruts et al.^47^ demonstrated both clinical recovery and rapid, durable restructuring of the recipient microbiota toward a donor-like community following transplantation. This early proof of microbiome restoration was followed by the landmark randomized trial by van Nood et al.^48^, in which donor fecal infusion achieved resolution of rCDI in 81% of patients after a single infusion and 94% after repeat treatment, compared with 31% with vancomycin alone and 23% with vancomycin plus bowel lavage. Subsequent evidence has confirmed high efficacy across different FMT protocols, with week-8 clinical cure rates of approximately 84% following a single FMT and 91% following repeated FMT.^49^ ^,^ ^50^ Similarly, pooled analyses have reported overall cure rates ranging from 70% to 91%, supporting the effectiveness of microbiota restoration strategies in rCDI.^51^

However, direct comparisons across studies and the reported cure rates should be interpreted cautiously because reported efficacy is influenced by considerable methodological and clinical heterogeneity. Representative studies illustrating differences in patient populations, delivery routes, follow-up durations, outcome definitions, and sustained cure rates are summarized in Table 1.

**Table 1.** Sources of heterogeneity influencing reported efficacy outcomes of FMT in recurrent CDI.

Population/Study characteristics	Delivery route	Follow-up duration	Outcome definition	Sustained cure rate/Main outcome	Key sources of heterogeneity	References
Patients with recurrent CDI included in systematic review and meta-analysis (45 studies)	Multiple routes (colonoscopy, enema, nasoenteric, capsules)	8 weeks	Clinical response	84% after single FMT; 91% after repeated FMT	Delivery route; repeat versus single FMT administration	Baunwall et al. [49]
Patients with recurrent CDI included in systematic review and meta-analysis (15 studies; *n* = 1,452)	Colonoscopy, tube delivery, capsules	Variable across studies	CDI recurrence prevention	Recurrence rate 16% after FMT vs. 42% with antibiotics	Administration route; study design; patient selection	Ray et al. [50]
Adults with recurrent CDI included in systematic review (7 studies; *n* = 1,030)	Colonoscopy and capsules; fresh/frozen donor material	Variable	Clinical cure	Cure rates ranged from 70% to 91%	Delivery route; donor material (fresh vs. frozen)	Weerakoon et al. [51]
First or second CDI; multicenter Danish cohort (*n* = 467)	Real-world FMT protocols	8 weeks	Sustained cure of C. difficile-associated diarrhea (CDAD)	55% after first FMT; 79% after repeated FMT	Repeat dosing strategy; disease severity; real-world setting	Paaske et al. [52]
CDI patients; multicenter real-world cohort (*n* = 1,170)	Capsules, colonoscopy, nasojejunal tube	8 weeks	Cure of CDAD	60% after first FMT; 81% after repeated FMT	Antibiotic pretreatment duration; delivery route; repeat FMT	Paaske et al. [52]
Ulcerative colitis with concomitant recurrent CDI (*n* = 35)	Single or repeated FMT	8 weeks	Negative C. difficile toxin/CDI cure	91% overall cure; repeated FMT superior to single FMT	Underlying inflammatory bowel disease; repeat dosing	Porcari et al. [53]
Recurrent CDI; randomized double-blind placebo-controlled trial (*n* = 153)	Oral FMT capsules	56 days	Recurrent diarrhea/CDI recurrence or death	No significant reduction versus placebo	Capsule formulation; endpoint definition; patient population	Drekonja et al. [54]
Adult recurrent CDI cohort (*n* = 240)	Colonoscopy	1 year	FMT failure (recurrent CDI or all-cause death)	24.6% failure rate; age ≥ 70 years, ≥ 4 prior CDI episodes, and diabetes associated with failure	Age; recurrence burden; diabetes mellitus	Nguyen et al. [55]
Recurrent CDI prospective cohort (*n* = 448)	Colonoscopic or oral administration	Post-FMT follow-up	Recurrence after antibiotic exposure	Post-FMT non-CDI antibiotics increased recurrence risk	Post-FMT antibiotic exposure; immunocompromised status	Hirsch et al. [56]
Elderly patients (≥85 years) with recurrent CDI (*n* = 43)	Colonoscopic FMT	8 weeks	CDI cure and safety	77% after first FMT; 88% overall efficacy after repeat FMT	Advanced age; frailty; comorbidity burden	Montalto et al. [57]
Adults with CDI treated by colonoscopy-based FMT (*n* = 103)	Colonoscopic FMT	2 months	Clinical success (formed stool or C. difficile-negative diarrhea)	87.3% clinical success	Donor source (family donor vs. stool bank)	Watts et al. [58]
Multi-cohort analysis of CDI and IBD FMT recipients (1,280 microbiota datasets)	Multiple FMT protocols	Longitudinal follow-up	Donor microbiota engraftment and FMT response	FMT outcome associated with donor–recipient microbiota matching	Recipient baseline microbiome; donor–recipient microbiota compatibility	He et al. [59]
rCDI patients receiving fresh, frozen, or lyophilized FMT formulations (*n* = 537)	Fresh, frozen, or lyophilized FMT	Variable storage duration	Clinical success after single-dose FMT	No significant efficacy differences between formulations; longer storage reduced efficacy	Stool preparation; formulation type; storage duration	Shaheen et al. [60]

As summarized in Table 1, the wide range of reported FMT efficacy estimates likely reflects substantial clinical and methodological heterogeneity rather than differences in treatment efficacy alone and therefore should be interpreted cautiously. Across studies, outcomes were influenced by factors related to FMT administration (delivery route, repeat dosing, donor source, and stool formulation), recipient characteristics (age, frailty, comorbidities, immune status, baseline microbiome composition, and recurrence burden), as well as differences in follow-up duration and outcome definitions. This variability is evident in real-world cohorts, where sustained cure rates have ranged from approximately 55% following a single FMT to nearly 80% after repeated administration, depending on treatment strategy and outcome definition.^52^ Similar findings have been reported in larger real-world populations, where prolonged antibiotic pretreatment and delivery through oral multi-dose capsules or colonoscopy were associated with improved outcomes.^61^

Differences in patient populations, disease severity, recurrence burden, donor characteristics, stool processing protocols, antibiotic pretreatment strategies, route of administration, number of FMT infusions, follow-up duration, and outcome definitions complicate direct comparisons between studies.^49‐51^

Emerging evidence suggests that donor-related factors, stool processing methods, and donor–recipient microbiome interactions represent additional sources of variability in FMT outcomes. Donor selection may influence treatment success, as stool obtained from family donors has been associated with a significantly higher risk of treatment failure compared with stool-bank donations, highlighting the importance of donor characteristics and screening procedures.^58^

Patient-specific factors further contribute to outcome variability. Recipient baseline microbiome composition may also affect therapeutic response, with donor–recipient microbiota matching and engraftment efficiency emerging as important determinants of successful colonization and clinical benefit following transplantation.^59^ High cure rates have been reported in patients with ulcerative colitis and concomitant rCDI, particularly following repeated FMT administration.^53^ Additional studies have identified advanced age, multiple prior CDI episodes, diabetes mellitus, immunocompromised status, and post-FMT antibiotic exposure as factors associated with treatment failure or recurrence.^55^ ^,^ ^56^ Nevertheless, favorable outcomes have also been observed in very elderly patients, suggesting that chronological age alone should not preclude consideration of FMT, although frailty may influence prognosis.^57^

Methodological differences in stool preparation and storage further contribute to heterogeneity across studies. Although fresh preparations have generally demonstrated the highest efficacy, frozen and lyophilized products retain substantial therapeutic effectiveness and offer important logistical advantages, with no significant differences consistently observed between fresh and frozen material in direct comparisons.^62^ Similarly, long-term storage of frozen donor stool has been shown to preserve microbial viability and maintain clinical effectiveness, supporting the feasibility of stool-bank–based FMT programs.^63^ More recently, analyses of different FMT formulations demonstrated that prolonged storage duration, rather than storage temperature alone, may negatively affect bacterial diversity and clinical efficacy, while advanced recipient age was also associated with less favorable outcomes.^60^ A randomized double-blind trial evaluating capsule-delivered FMT did not demonstrate a significant reduction in recurrence or death compared with placebo, highlighting the influence of formulation on reported efficacy.^54^

Together, these findings highlight the multifactorial nature of FMT efficacy and underscore the need for greater standardization of efficacy endpoints, patient stratification, donor selection, microbiome characterization, manufacturing procedures, and product storage conditions.^64^

#### 3.1.2. Standardized microbiota-based and LBPs

The transition from conventional FMT toward standardized microbiota-based therapeutics represents an important step in the clinical development of microbiome restoration strategies. These interventions encompass a heterogeneous spectrum, ranging from donor-derived whole-microbiota products that retain conceptual similarities to FMT to more compositionally defined microbial preparations and FDA-approved LBPs, which are administered following completion of standard antibiotic treatment to reduce recurrence risk through microbiome restoration. Unlike antibiotics, these products aim to re-establish colonization resistance by restoring key microbial functions and ecological networks disrupted during CDI and its treatment.^65^ ^,^ ^66^

Although these approaches share the objective of restoring colonization resistance, they differ substantially in microbial complexity, manufacturing, formulation, delivery, and degree of compositional control.^67^

This distinction is particularly relevant to the two microbiota-based therapeutics approved by the FDA for the prevention of recurrent CDI. Despite sharing a common goal of microbiome restoration, REBYOTA and VOWST differ substantially in composition, manufacturing, processing, and delivery, illustrating distinct approaches and levels of refinement and standardization within the broader transition from conventional FMT toward regulated microbiota-based therapeutics. Fecal microbiota, live-jslm (REBYOTA™, 2022) is a donor-derived, whole-microbiota product administered rectally, which is more appropriately considered within the continuum of standardized FMT-derived therapies rather than as a defined LBP. In contrast, live-brpk (VOWST™, 2023), consists of a highly purified, orally administered *Firmicutes* spore preparation derived from donor stool administered orally.^65^ ^,^ ^66^

Clinical evidence supporting REBYOTA™ is primarily derived from the PUNCH trial program, which demonstrates a favorable but heterogeneous efficacy profile. While early open-label and historically controlled studies suggested high response rates, up to 80–85%, including in high-risk populations, randomized controlled data yielded more modest and variable outcomes, with only the PUNCH-CD3 trial achieving regulatory endpoints using a Bayesian borrowing framework.^66,68‐71^ These findings indicate that although REBYOTA™ confers a measurable therapeutic benefit, its efficacy is sensitive to study design, dosing strategy, and patient selection. More recent analyses integrating seven clinical studies and more than 1,700 participants further support its efficacy across diverse adult populations and clinical settings.^72^

VOWST™ (fecal microbiota spores, live-brpk; formerly SER-109) represents a compositionally more refined donor-derived microbiota product. In the phase 3 ECOSPOR III trial, CDI recurrence through 8 weeks occurred in approximately 12% of VOWST-treated participants compared with 40% receiving placebo.^65^ Mechanistic studies of VOWST support engraftment of *Firmicutes* species and restoration of microbiome-associated metabolic functions, including bile acid metabolism, as potential contributors to colonization resistance.^73^ ^,^ ^74^ Importantly, restoration of bile acid profiles is not unique to VOWST and has also been observed following RBX2660 administration, with reductions in primary bile acids and increases in secondary bile acids sustained during follow-up.^75^

Beyond approved products, several investigational microbiota-based therapeutics further illustrate both the promise and the challenges of this evolving therapeutic landscape. RBX7455, an oral, lyophilized derivative of RBX2660, and CP101, a single-dose donor-derived capsule, have shown encouraging efficacy and safety signals but remain constrained by limited controlled data and regulatory complexities.^76^ ^,^ ^77^ Notably, the discontinuation of CP101 due to manufacturing and regulatory challenges, rather than lack of efficacy, underscores the importance of scalable production and standardized quality control in microbiome therapeutics.^78‐80^

These findings highlight a central paradox in microbiome-based therapy: while broad microbial restoration, as achieved by FMT, yields high efficacy, increasing precision and standardization may introduce trade-offs in therapeutic robustness. LBPs attempt to balance these competing priorities by defining microbial composition while preserving key ecological functions, yet clinical outcomes remain sensitive to dosing, formulation, and host-specific factors.

Thus, the progressive refinement and standardization of microbiota-based therapeutics represent an important step toward the rational design of microbiome interventions, bridging empirical ecological restoration and more compositionally defined therapeutic strategies. Their continued development will depend on improved mechanistic understanding, harmonized clinical trial design, and scalable manufacturing strategies that ensure both safety and reproducibility. A comparative overview of FDA-approved and investigational microbiota-based therapeutics, including their composition, clinical performance, and development status, is presented in Table 2.

**Table 2.** FDA-approved and investigational microbiota-based therapeutics for rCDI.

Product	Type	Composition	Administration	Clinical Evidence	Efficacy (recurrence/success)	Limitations	Status	References
REBYOTA™ (RBX2660)	Whole-microbiota/FMT-derived standardized product	Whole-stool derived microbiota	Rectal (enema)	PUNCH trials (I–III), real-world study	~78–83% success (real-world ~83%)	Variable efficacy; dependence on study design; non-standardized composition	FDA approved (2022)	[68‐71,81]
VOWST™ (SER-109)	Spore-based LBP	Purified Firmicutes spores (~1% donor microbiota)	Oral capsules	ECOSPOR trials (Phase 1–3), systematic reviews	~12% recurrence vs 40% placebo; ~8.7% real-world	Early heterogeneity; dose sensitivity; diagnostic variability	FDA approved (2023)	[65,73,74,82,83]
RBX7455	Oral microbiota LBP	Lyophilized derivative of RBX2660	Oral	Early-phase open-label study	~10–20% recurrence	No controlled trials; small cohorts	Early development	[77]
CP101	Donor-derived microbiota capsule	Whole microbiota (oral capsule)	Oral (single dose)	PRISM3 Phase 2 trial	74.5% recurrence-free vs 61.5% placebo (8 weeks)	Regulatory/manufacturing issues; halted	Discontinued	[80]

#### 3.1.3. Defined microbial consortia

Defined microbial consortia represent a rational evolution of additive microbiome-based therapies, aiming to reproduce the ecological benefits of FMT using controlled, well-characterized microbial communities. Unlike whole microbiota transfer, these consortia consist of selected bacterial strains with defined functional properties, enabling greater reproducibility, mechanistic clarity, and regulatory compatibility.^84^ ^,^ ^85^

In the context of CDI, defined consortia are designed to restore key ecological functions underlying colonization resistance, including short-chain fatty acid production, bile acid metabolism, and competitive nutrient utilization. These functions are often disrupted following antibiotic exposure and are only partially or inconsistently reconstituted by conventional therapies.^86^ By targeting these pathways, synthetic consortia aim to provide a more precise and scalable alternative to FMT.

VE303 represents one of the most advanced examples of this approach. Composed of eight commensal clostridial strains, this consortium has demonstrated dose-dependent efficacy in reducing recurrent CDI in phase 2 trials, alongside evidence of rapid microbiome engraftment and metabolic restoration.^87‐89^ However, as with other microbiome-based interventions, clinical outcomes remain influenced by host variability and study design, and larger trials are required to confirm therapeutic consistency.

Increasing evidence indicates that successful microbial consortia operate through multiple complementary ecological mechanisms rather than the activity of individual strains. Defined communities have been shown to reduce pathogen-associated *Proteobacteria*, replenish obligate anaerobes and butyrate-producing bacteria, and decrease antimicrobial resistance gene burdens, producing microbiome-wide effects comparable to those observed after FMT.^90^ Mechanistic studies further demonstrate that nutrient competition is a major determinant of colonization resistance. Targeted microbial communities capable of consuming host-derived mucosal sugars can restrict access of *C. difficile* to key nutrients required for expansion,^91^ while inflammation-driven alterations in nutrient availability may further shape pathogen persistence and competitive interactions within the gut ecosystem.^92^

Foundational studies established the mechanistic link between bile acid metabolism and *C. difficile* pathogenesis. Sorg and Sonenshein first demonstrated that specific bile salts regulate *C. difficile* spore germination, with cholate derivatives acting as germinants in the presence of glycine.^93^ Subsequent work showed that antibiotic-induced alterations in the intestinal microbiota modify bile salt metabolism and create conditions favoring *C. difficile* spore germination in vivo.^94^ Extending these observations, Theriot et al.^95^ linked antibiotic-mediated depletion of secondary bile acids with increased susceptibility to *C. difficile* germination and outgrowth, while Buffie et al.^96^ identified the bile acid 7α-dehydroxylating bacterium *Clostridium scindens* as a contributor to colonization resistance.

Secondary bile acid metabolism contributes to pathogen suppression; however, colonization resistance cannot be explained by bile acid metabolism alone. In a murine model, Aguirre et al.^97^ demonstrated that bacteria capable of 7α-dehydroxylating primary bile acids protected against *C. difficile* even when secondary bile acid generation was absent, providing evidence that protection can occur through bile acid-independent mechanisms involving competition for nutrients required for *C. difficile* growth. These findings, together with more recent evidence, indicate that colonization resistance reflects the combined activity of multiple microbial metabolic pathways rather than secondary bile acid production alone.^97‐99^ In particular, competition for amino acids involved in Stickland fermentation, especially proline utilization, has emerged as an important determinant of *C. difficile* fitness and persistence.^99^ ^,^ ^100^ Community-scale metabolic modelling further supports the concept that susceptibility to *C. difficile* colonization is governed by complex microbiota-wide metabolic interactions involving metabolites such as succinate, trehalose, and ornithine rather than by individual taxa alone.^101^

Beyond predefined strain combinations, advances in computational biology and synthetic ecology have enabled the design of function-driven microbial communities. Machine learning–guided approaches have identified minimal consortia capable of reproducing key protective effects of FMT. For example, a 37-strain synthetic community suppressed *C. difficile in vitro* and in gnotobiotic mouse models, while further reduction to a single proline-fermenting strain retained substantial activity, supporting the concept of function-based therapeutic design that requires validation in human studies.^102^ Nevertheless, caution is warranted when extrapolating these findings to human disease. Studies using synthetic microbial ecosystems have demonstrated that even taxonomically and functionally defined communities may fail to fully reproduce colonization resistance, suggesting that ecological redundancy, interspecies cooperation, and host–microbiome interactions remain critical for durable protection against CDI.^103^

Defined microbial consortia bridge the gap between empirical microbiome restoration and precision microbiome engineering. By enabling targeted reconstruction of key ecological functions, they represent a promising next-generation therapeutic strategy for CDI.

Despite important advances in the field, several challenges remain. Defined consortia must balance ecological complexity with manufacturability, as excessive simplification may compromise resilience and functional redundancy, whereas increasing complexity may reintroduce variability and regulatory challenges. In addition, translating findings from controlled experimental systems to the heterogeneous human gut environment remains a major obstacle. Therefore, further work is needed to optimize consortium composition, dosing strategies, and patient selection while preserving the ecological stability required for long-term colonization resistance.

### 3.2. Subtractive therapy

Subtractive therapy encompasses strategies aimed at selectively eliminating *C. difficile* while preserving the functional integrity of the gut microbiome. Unlike broad-spectrum antimicrobial approaches, these strategies focus on pathogen-specific suppression or disruption of key survival mechanisms, thereby reducing recurrence risk and limiting collateral ecological damage.^104^ Four principal modalities have emerged within this framework: targeted antibiotics, bacteriocins, bacteriophages and endolysins, and CRISPR-based antimicrobials.

#### 3.2.1. Targeted antibiotics

Targeted antibiotic therapy remains the cornerstone of CDI management and represents the most clinically established subtractive strategy. Current first-line agents, including oral vancomycin and fidaxomicin, effectively suppress *C. difficile* while differing substantially in their impact on the gut microbiome.^105^ ^,^ ^106^ Fidaxomicin, a narrow-spectrum macrocyclic antibiotic, has demonstrated a more favorable ecological profile than vancomycin by preserving commensal taxa and maintaining secondary bile acid metabolism, key determinants of colonization resistance. Consistent randomized clinical trial data support its use as the preferred agent for both primary and recurrent CDI, with improved sustained cure rates and reduced recurrence risk.^107‐109^ Recent guideline updates and real-world studies further reinforce fidaxomicin as the preferred first-line therapy for non-fulminant CDI, particularly in patients at increased risk of recurrence, while vancomycin remains an effective alternative when fidaxomicin is unavailable or contraindicated.^110‐112^

For recurrent CDI, treatment strategies increasingly extend beyond pathogen eradication alone. Vancomycin taper-and-pulse regimens remain an accepted therapeutic option in selected patients and continue to be incorporated into contemporary treatment algorithms. In a retrospective cohort study, tapered vancomycin regimens were associated with lower recurrence rates than standard vancomycin therapy, although prospective randomized studies are required to confirm these findings.^113^ Recent randomized clinical trial data have further evaluated vancomycin pulse-and-taper strategies for recurrent CDI, although their optimal role relative to newer microbiome-based interventions remains under investigation.^114^

Conventional antibiotics remain imperfect, as recurrence rates remain substantial and microbiome disruption persists, particularly with vancomycin-based regimens. These limitations have driven the development of next-generation microbiome-sparing antibiotics designed to preserve ecological function while maintaining anti-*C. difficile* activity.

Ridinilazole represents one of the most advanced candidates in this class. Early-phase studies demonstrated superior sustained clinical response compared with vancomycin, reduced recurrence rates, and minimal impact on microbial diversity.^115^ However, phase 3 trials yielded more nuanced results, showing comparable overall clinical response but a significant reduction in recurrence and improved preservation of bile acid metabolism.^116^ These findings suggest that traditional clinical endpoints may underestimate the therapeutic value of microbiome-preserving agents. Indeed, emerging analyses highlight the need for microbiome-informed outcome measures that better capture sustained ecological recovery.^117^

Ibezapolstat, a first-in-class DNA polymerase IIIC inhibitor, further exemplifies this shift toward selective antimicrobial targeting. Phase 2 data indicate clinical cure rates comparable to vancomycin while preserving microbial diversity and beneficial commensal taxa.^118^ ^,^ ^119^ Similarly, in preclinical models of recurrent CDI, the experimental glycopeptide EVG7 demonstrated potent anti-*C. difficile* activity while preserving key commensal populations, including members of the *Lachnospiraceae* family, suggesting a potential advantage over broader-spectrum antimicrobial approaches.^120^ These agents illustrate a transition from pathogen eradication toward ecological modulation, in which therapeutic success is increasingly defined not only by bacterial clearance but also by preservation or restoration of microbiome functionality. Nevertheless, even the most selective antibiotics remain inherently reductive, targeting the pathogen without directly rebuilding disrupted microbial communities.

Management of fulminant CDI differs substantially from that of non-fulminant disease. Current guidelines recommend high-dose oral or nasogastric vancomycin combined with intravenous metronidazole, with rectal vancomycin administration considered when ileus is present. In selected refractory cases, additional agents such as tigecycline may also be considered.^110^ In this setting, rapid pathogen control and prevention of life-threatening complications remain the primary therapeutic priorities.^77^ ^,^ ^105^

Overall, targeted antibiotics serve as both essential frontline therapies and transitional interventions that stabilize the microbial ecosystem prior to restorative treatment. Their continued evolution toward microbiome-sparing designs reflects a broader paradigm shift in CDI management, emphasizing ecological preservation alongside pathogen control.

#### 3.2.2. Bacteriocins

Bacteriocins are included in the category of postbiotics, defined as preparations of inanimate microorganisms and/or their components that confer a health benefit on the host, may provide a means of delivering microbiota-derived bioactive compounds.^121^ Bacteriocins represent a highly selective subtractive strategy, offering targeted inhibition of *C. difficile* with minimal disruption of commensal microbiota. These ribosomally synthesized antimicrobial peptides exert their activity through diverse mechanisms, including membrane permeabilization, induction of cell lysis, inhibition of vegetative growth, interference with toxin-associated processes, and suppression of spore germination or outgrowth.^122^ ^,^ ^123^ Unlike conventional antibiotics, their narrow-spectrum activity positions them as promising candidates for microbiome-sparing therapy.

Several bacteriocins act primarily through direct antimicrobial effects on vegetative *C. difficile* cells. Among these, nisin has demonstrated strong inhibitory activity in *ex vivo* colonic models, achieving complete suppression of *C. difficile* at defined concentrations while inducing only modest, dose-dependent alterations in the surrounding microbial community.^124^ Together, these findings highlight the potential of bacteriocins to achieve pathogen control while preserving key components of the intestinal ecosystem.

Mechanistic differences among bacteriocins may translate into distinct ecological consequences. In a recent preclinical study employing in vitro susceptibility testing and ex vivo fecal community models, thuricin CD demonstrated highly selective activity directed primarily against *C. difficile*, whereas nisin exerted broader antimicrobial effects across Gram-positive members of the gut microbiota. These observations suggest that not all bacteriocins offer the same degree of microbiome preservation and reinforce the need to evaluate ecological outcomes alongside antimicrobial efficacy when developing bacteriocin-based therapies for CDI.^125^

Beyond inhibition of vegetative growth, certain bacteriocins may influence additional stages of the pathogen life cycle. In this context, a postbiotic preparation containing bacteriocin-like molecules, including enterolysin A, demonstrated *in vitro* inhibitory activity against *C. difficile* vegetative cells and spore germination.^126^ Preliminary *in vitro* evidence also suggests that bacteriocin-based approaches may benefit from combination strategies. For example, durancin 61 A exhibited synergistic activity with reuterin against *C. difficile*, whereas bacteriocin-producing bacterial isolates demonstrated inhibitory activity against clinical *C. difficile* strains, supporting further evaluation of these approaches in preclinical models.^127^ ^,^ ^128^ These findings indicate that bacteriocins may contribute to CDI control through multiple complementary mechanisms and could potentially be incorporated into combination-based therapeutic strategies.

However, bacteriocin efficacy appears highly dependent on ecological context. While certain bacteriocin-producing strains may promote colonization resistance, others may exert unintended effects on microbiome recovery and ecosystem stability.^129^ ^,^ ^130^ Microbial interactions within complex gut communities can substantially modify antimicrobial susceptibility and therapeutic outcomes, making the effects of bacteriocin-based interventions difficult to predict outside the native microbiome environment.^131^ Recent experimental evidence further illustrates this complexity. In a murine model, a lantibiotic-producing Blautia pseudococcoides strain altered microbiome recovery following antibiotic-induced dysbiosis and was associated with sustained reductions in colonization resistance against *C. difficile*, highlighting that microbiome-targeted antimicrobial strategies may influence broader ecosystem dynamics beyond direct pathogen inhibition.^132^ These findings underscore a key translational challenge: therapeutic efficacy depends not only on anti-*C. difficile* activity but also on preservation of microbiome resilience and ecological function.

Preclinical studies further support the potential of bacteriocins to suppress multidrug-resistant *C. difficile* while preserving microbial diversity.^133^ ^,^ ^134^ Nevertheless, successful clinical translation will also depend on overcoming pharmacokinetic and formulation-related limitations. Several bacteriocins exhibit restricted stability, solubility, and gastrointestinal persistence, which may limit their performance *in vivo*. To address these challenges, delivery strategies such as nanoformulations and encapsulation systems are being investigated. For example, liposomal encapsulation improved the stability and bioactivity of thuricin CD in preclinical studies, supporting further evaluation of formulation-based approaches for targeted colonic delivery.^135^

Overall, bacteriocins exemplify the promise of precision subtractive therapy by combining antimicrobial activity with varying degrees of ecological selectivity. However, their future clinical utility will depend not only on antimicrobial potency but also on a deeper understanding of host–microbiome interactions, ecological context, and the development of effective delivery systems capable of maintaining activity within the gastrointestinal environment.

#### 3.2.3. Bacteriophages & endolysins

Bacteriophages represent an emerging subtractive strategy capable of selectively targeting *C. difficile* while preserving the surrounding microbiota. By infecting specific bacterial hosts, phages enable precision reduction of pathogen load and can be tailored to target virulent ribotypes associated with disease severity.^136^ ^,^ ^137^ However, their therapeutic application is constrained by several important biological limitations. A major obstacle specific to *C. difficile* is the lack of naturally occurring strictly lytic phages suitable for therapeutic use, as characterized *C. difficile* phages are predominantly temperate and therefore retain the potential for lysogeny. This may compromise bacterial killing, promote lysogen formation, and raises concerns regarding horizontal gene transfer, making temperate phages less suitable for conventional phage therapy. Additional limitations include narrow host range, emergence of phage resistance, and dependence on actively replicating bacterial cells.^138^ ^,^ ^139^

Phage cocktails partially address these challenges by expanding host coverage and improving ecological stability. Current evidence for phage therapy in CDI remains predominantly preclinical. Studies using *in vitro* gut fermentation models and experimental infection systems have shown that multi-phage formulations can markedly reduce *C. difficile* abundance while largely preserving commensal bacterial populations, supporting the rationale for further translational development.^140^ ^,^ ^141^

Preclinical studies suggest that phage-derived endolysins may represent a versatile alternative to whole-phage therapy. These enzymes directly degrade the bacterial cell wall independently of phage replication, enabling rapid bacteriolytic activity against *C. difficile*. Experimental studies have shown that several lysins, including PlyCD, CD27L, and LysCD6356, can lyse diverse *C. difficile* strains, including hypervirulent ribotypes, while some candidates have also demonstrated activity against spore outgrowth or preserved overall microbiota diversity in model systems.^142‐144^ Importantly, engineered variants exhibit improved stability, enhanced lytic potency, and selective activity within complex microbial communities, as well as synergistic effects when combined with conventional antibiotics. However, current evidence remains largely restricted to *in vitro*, *ex vivo*, and animal studies, and clinical validation is still lacking.

The advantages of lysin-based strategies are further highlighted by their capacity to target multiple stages of the *C. difficile* lifecycle. For example, the PlyCD–HD5 fusion protein combines phage-derived lytic activity with a host antimicrobial peptide and, in murine CDI models, was associated with reduced toxin production, decreased sporulation, and improved survival outcomes.^145^ These properties distinguish lysins from conventional phage therapy, which primarily targets actively replicating cells and does not directly address spore-mediated recurrence. Although these findings highlight the potential of rationally engineered lysins, their therapeutic utility remains to be validated in human studies.

Advances in synthetic biology are also enabling the development of engineered phages with enhanced antibacterial performance. For example, CRISPR–Cas3–armed phages have demonstrated improved killing efficiency and the ability to overcome natural host-range limitations, offering a hybrid approach that combines phage specificity with programmable genetic targeting.^146^

Overall, while phage-based strategies provide highly specific pathogen targeting and ecological compatibility, their clinical translation remains constrained by the lack of naturally occurring strictly lytic *C. difficile* phages, narrow host range, lysogeny, and emergence of phage resistance. Endolysins, by contrast, offer more consistent efficacy, broader activity, and greater mechanistic flexibility, positioning them as the most promising phage-derived approach for CDI. However, the absence of large-scale clinical studies remains a major barrier to translation, and further work is required to optimize delivery, dosing, and integration with existing therapeutic strategies.

#### 3.2.4. CRISPR-based antimicrobials

CRISPR-based antimicrobials represent a new generation of precision subtractive therapies, enabling sequence-specific targeting of *C. difficile* at the genomic or transcriptomic level. By directing CRISPR-associated nucleases toward essential genes, toxin loci such as tcdA and tcdB, or antibiotic resistance determinants, these systems can selectively eliminate pathogenic strains while preserving commensal microbiota.^147^

A key advantage of CRISPR-based approaches lies in their versatility of targeting and delivery. CRISPR–Cas systems, including Cas9, Cas13a, and Cpf1, can be delivered using engineered bacteriophages, conjugative plasmids, or nanoparticle-based platforms, allowing both DNA and RNA targeting strategies.^148‐150^ These tools enable not only direct bacterial killing but also functional modulation, such as suppression of toxin production, reduction of sporulation, or removal of mobile genetic elements that contribute to virulence.

Experimental investigations have demonstrated that CRISPR-engineered phages can enhance antibacterial efficacy beyond that of their wild-type counterparts, including improved killing of target bacteria, activity in biofilm-associated settings, and reduced emergence of resistant variants *in vitro* and in animal models.^146^ ^,^ ^151^ More recently, engineered phage platforms incorporating CRISPR-guided transposases have enabled targeted genome editing within microbial communities, illustrating the potential to manipulate bacterial functions and community composition rather than simply eliminating pathogens.^152^ Although these advances highlight the versatility of programmable antimicrobials, their safety, delivery efficiency, ecological consequences, and clinical utility remain to be established in human studies.

Despite these promising advances, CRISPR-based antimicrobials remain at an early stage of development. Challenges related to delivery efficiency, off-target effects, horizontal gene transfer, and immune responses must be addressed before clinical translation. In addition, most current evidence derives from *in vitro* and preclinical models, with limited data in human systems. Experience from non-CDI applications, such as CRISPR-based phage therapy for antibiotic-resistant *Escherichia coli*, further underscores both the therapeutic potential and the complexity of implementing these technologies *in vivo.* ^153^

Overall, CRISPR-based antimicrobials represent the most precise and programmable form of subtractive therapy, with the capacity to selectively eliminate, attenuate, or re-engineer pathogenic bacteria. However, their clinical application will depend on overcoming key translational barriers and integrating these tools within broader microbiome-targeted therapeutic frameworks. A comparative overview of subtractive therapeutic strategies is presented in Table 3.

**Table 3.** Overview of subtractive microbiome-based strategies in CDI.

Strategy	Mechanism	Specificity	Microbiome impact	Advantages	Limitations	Development stage
Targeted antibiotics	Inhibition of bacterial growth (e.g., DNA/RNA synthesis)	Moderate	Variable (fidaxomicin > vancomycin)	Clinically validated; rapid effect	Recurrence; residual dysbiosis	Clinical standard
Bacteriocins	Membrane disruption, toxin interference	High	Minimal	Narrow-spectrum; ecological preservation	Context-dependent efficacy	Preclinical
Phages	Infection and lysis of specific strains	Very high	Minimal	Strain-specific targeting	Resistance; narrow host range	Preclinical
Endolysins	Enzymatic cell wall degradation	High	Minimal	Broad strain activity; rapid killing	Delivery challenges	Preclinical
CRISPR antimicrobials	Sequence-specific DNA/RNA targeting	Ultra-high	Minimal	Programmable; precision targeting	Delivery; off-target effects	Early experimental

### 3.3. Modulatory therapy

Modulatory therapy encompasses a diverse set of interventions that do not directly eliminate *C. difficile*, but instead target the ecological and host–pathogen mechanisms that enable colonization, toxin activity, and recurrence. In contrast to additive strategies, which restore microbial communities, and subtractive strategies, which suppress the pathogen, modulatory approaches reshape the gut environment or host responses to reduce virulence and re-establish colonization resistance.^154^

Conceptually, modulatory interventions can be grouped into three major categories: passive immunization, active immunization, and host–microbiota modulation. These strategies act at different levels of the disease process, including toxin neutralization, inhibition of spore germination, and reinforcement of mucosal and immune defenses.

*Passive immunization* provides immediate protection through the administration of pre-formed neutralizing molecules, such as monoclonal antibodies targeting *C. difficile* toxins. Bezlotoxumab provided clinical proof of concept for passive immunization against CDI by demonstrating that toxin B neutralization could reduce recurrence.^155^ Next-generation passive immunization platforms, including IgY antibodies, VHH nanobodies, and engineered probiotic delivery systems, aim to improve specificity, stability, and localized activity within the gut.^156‐158^

In contrast, *active immunization* strategies stimulate endogenous immune responses against toxin-derived antigens, generating durable systemic and mucosal protection. Vaccine-based approaches, including toxoid formulations and recombinant vectors, are designed to prevent toxin-mediated damage and interrupt early stages of infection.

Host–microbiota modulation targets the metabolic and ecological conditions that facilitate *C. difficile* persistence. Key approaches include modulation of bile acid metabolism, inhibition of spore germination pathways, and restoration of microbial functions that support colonization resistance. Additional strategies aim to strengthen mucosal immunity and epithelial barrier integrity through host-directed immunological pathways and microbiota-derived metabolites.

Together, these modalities represent an ecological and host-oriented therapeutic framework that complements additive and subtractive approaches. By targeting the underlying mechanisms of recurrence rather than the pathogen itself, modulatory therapies offer a promising avenue for next-generation interventions in CDI and other dysbiosis-driven diseases.

#### 3.3.1. Passive immunization

Passive immunization provides immediate protection by delivering pre-formed neutralizing molecules that target *C. difficile* toxins or early colonization pathways. Unlike vaccines, these interventions do not activate the host immune response and therefore achieve rapid therapeutic effects, particularly in high-risk or immunocompromised patients.^159‐161^

In CDI, passive immunization primarily targets neutralization of toxins A and B, the key drivers of epithelial damage and disease recurrence. Among these approaches, monoclonal antibodies represent the most clinically advanced strategy, while emerging platforms, including nanobodies and engineered delivery systems, aim to enhance specificity and local activity within the gut.

##### 3.3.1.1. Anti-toxin monoclonal antibodies

Monoclonal antibodies represent the most clinically validated form of passive immunotherapy in CDI, providing direct neutralization of the major virulence factors, toxins A (TcdA) and B (TcdB).^162‐164^ By targeting toxin-mediated epithelial damage rather than bacterial burden, these agents reduce disease severity and recurrence without disrupting the gut microbiome.

Bezlotoxumab, a fully human monoclonal antibody targeting toxin B, previously represented an adjunctive option for patients at increased risk of CDI recurrence, including older adults, immunocompromised individuals, and those with previous CDI episodes, providing an important clinical proof of concept for toxin-targeted passive immunization. Clinical and real-world studies demonstrated reduced recurrence when bezlotoxumab was administered alongside standard antibiotic therapy, particularly in selected high-risk populations.^165‐167^ However, its manufacturer discontinued Zinplava in January 2025, and bezlotoxumab is therefore no longer available for routine clinical use in the United States.^155^ Consequently, questions regarding its optimal positioning within contemporary CDI treatment algorithms are now primarily of historical and mechanistic relevance.

Its efficacy was established in the pivotal MODIFY I and II randomized trials, where it achieved an absolute reduction of approximately 10–12% in recurrence compared with placebo in a cohort exceeding 2,600 patients, with a safety profile comparable to placebo.^168^ Notably, the addition of actoxumab (anti-TcdA) did not provide additional benefit, reinforcing the central role of toxin B in CDI pathogenesis.

Subsequent real-world investigations have extended these findings to broader and more clinically complex populations. Observational cohorts and prospective multicenter studies have reported lower recurrence rates following bezlotoxumab administration in patients at increased risk of rCDI, including older adults, immunocompromised individuals, and patients with multiple recurrence risk factors. Across these studies, recurrence rates generally ranged from approximately 6% to 17%.^165^ ^,^ ^169^ ^,^ ^170^ While these findings support the effectiveness of bezlotoxumab beyond randomized clinical trial settings, the available evidence remains largely observational and should therefore be interpreted with appropriate caution.

Combination strategies have also been explored. In a retrospective real-world study, the combination of fidaxomicin and bezlotoxumab was associated with higher global and clinical cure rates than fidaxomicin alone and showed a trend toward lower recurrence rates, suggesting that bacterial suppression and toxin neutralization may exert complementary therapeutic effects in CDI management.^171^ Similarly, observational studies have reported favorable sustained clinical response rates when bezlotoxumab is administered at the end of standard antibiotic treatment, although the optimal timing of administration remains to be established.^172^

Mechanistic studies provide additional insight into the benefits of toxin neutralization. Beyond preventing epithelial injury, anti-TcdB antibodies have been shown to mitigate systemic toxic effects and preserve immune homeostasis, including maintenance of innate immune responses and mucosal immune cell populations.^158^ ^,^ ^173^ These findings suggest that monoclonal antibodies modulate disease progression without impairing protective host immunity.The clinical experience with bezlotoxumab provides important proof of concept that selective toxin B neutralization can reduce CDI recurrence and continues to inform the development of next-generation passive immunization strategies. Because monoclonal antibodies do not directly restore microbiome composition, future approaches may benefit from integration with microbiome-restorative therapies and from alternative antibody formats and delivery systems. Preclinical studies using viral vector–mediated expression of anti-toxin antibodies have demonstrated sustained protective levels and complete protection in animal models, suggesting a potential strategy for long-term prophylaxis without repeated dosing.^163^ ^,^ ^174^

##### 3.3.1.2. VHH single-domain nanobodies

VHH nanobodies are small, highly stable single-domain antibody fragments derived from camelids that have emerged as flexible and potent toxin-neutralizing agents in CDI. Compared with conventional monoclonal antibodies, VHHs exhibit enhanced stability, reduced molecular size, and improved tissue penetration, making them particularly suitable for targeting toxins within the gastrointestinal environment. Anti-TcdA and anti-TcdB nanobodies have demonstrated the ability to neutralize toxin activity, prevent epithelial cytotoxicity, and confer protection across multiple *in vitro* and *in vivo* infection models.^145^ ^,^ ^156^ ^,^ ^175^ Structural studies further reveal diverse mechanisms of toxin neutralization, including stabilization of the glucosyltransferase domain and interference with conformational changes required for pore formation.^176^ These findings highlight the versatility of VHH-based inhibition strategies and support their development as highly specific anti-toxin agents.

A key advantage of nanobody platforms lies in their modular architecture, which enables straightforward engineering of multivalent and bispecific constructs with enhanced neutralizing potency. In addition, their high robustness allows integration into innovative delivery systems, including genetically engineered probiotic strains capable of secreting functional nanobodies directly within the gut lumen.^177^ Viral vector–mediated delivery approaches have also demonstrated sustained in vivo expression and durable protection against CDI in preclinical models, suggesting a potential route toward long-term toxin neutralization.^156^

Beyond VHH nanobodies, alternative compact biologic scaffolds such as designed ankyrin repeat proteins (DARPins) have emerged as promising toxin-binding platforms. These engineered molecules exhibit high affinity and specificity for TcdB, in some cases surpassing the neutralizing capacity of conventional monoclonal antibodies, while retaining stability under gastrointestinal conditions. Recent developments have also produced protease-resistant variants capable of maintaining activity following oral administration, supporting their potential as orally deliverable therapeutics.^175^ ^,^ ^178^

Complementary approaches include IgY-based antibodies, which represent a non-antibiotic, orally administered strategy targeting toxin activity or spore adhesion. Preclinical studies have demonstrated that anti-spore IgY significantly reduces epithelial adherence and delays disease onset in murine CDI models, while also enhancing the efficacy of standard antibiotic therapy.^157^ ^,^ ^179^

These next-generation antibody platforms expand the scope of passive immunization by offering improved stability, flexible engineering, and alternative delivery routes. Although still largely in preclinical development, they represent a promising direction for precision, microbiome-sparing interventions in CDI.

#### 3.3.2. Active immunization

Active immunization strategies in CDI aim to induce durable protective immunity against key virulence determinants, particularly toxins A (TcdA) and B (TcdB), with the goal of reducing toxin-mediated disease and recurrence. Most clinical vaccine candidates have focused on toxoid-based formulations, which have consistently demonstrated favorable safety profiles and robust immunogenicity in humans.^180^

Phase 2 studies showed that bivalent toxoid vaccines elicited strong and persistent neutralizing antibody responses against both toxins, particularly at higher doses, with immunity maintained for several years and further enhanced by booster administration.^181‐183^ However, these encouraging immunological outcomes did not translate into clinical efficacy. Large phase 3 trials failed to demonstrate protection against primary CDI and were terminated early because of futility despite favorable safety and immunogenicity profiles.^184^ ^,^ ^185^

This apparent disconnect between immunogenicity and protection highlights an unresolved question in toxin-centered vaccine strategies. Although toxoid vaccines consistently induced robust systemic neutralizing antibody responses, it remains unclear whether their lack of clinical efficacy primarily reflected insufficient toxin-neutralizing activity at the intestinal mucosa or whether toxin neutralization alone is intrinsically insufficient to prevent CDI. Serum antibody responses may not accurately represent the magnitude, localization, or functional activity of antibodies at the intestinal mucosal surface, where *C. difficile* toxins exert their pathogenic effects. At the same time, toxin-directed vaccines act predominantly downstream of colonization and therefore do not directly prevent earlier pathogenic events, including spore germination, intestinal colonization, microbiota disruption, and ecological niche establishment. Thus, the failure of phase 3 toxoid vaccines should not be interpreted as evidence that toxin-neutralizing immunity is biologically irrelevant, but rather that systemic antitoxin responses may represent an incomplete correlate of protection.^186^

Host-related factors may further contribute to the discrepancy between vaccine immunogenicity and clinical protection. Older adults and individuals with substantial comorbidity burden, who represent important target populations for CDI vaccination, may have reduced vaccine responsiveness because of immunosenescence and underlying immune dysfunction. Baseline antitoxin immunity may also influence vaccine responsiveness, as individuals with pre-existing anti-TcdA or anti-TcdB antibodies appear more likely to develop stronger post-vaccination responses, potentially reflecting prior exposure and memory B-cell priming.^187^ Together, these observations indicate that serum antitoxin antibody responses alone may not adequately capture the immune correlates required for protection against CDI.

Recognition of these limitations has stimulated a shift toward multivalent vaccine strategies that extend antigenic coverage beyond toxins alone. Recent preclinical approaches have combined toxin-derived antigens with targets associated with *C. difficile* spores and vegetative cells, thereby addressing both toxin-mediated injury and earlier stages of pathogen colonization and persistence. In the murine study by Thomas et al.^188^, the vaccine formulation incorporated toxin-derived epitopes together with spore- and vegetative-cell-associated antigens. Mucosal vaccination generated fecal antibody responses and TH17-associated tissue-resident immunity and was associated with enhanced pathogen clearance, protection against recurrence, and sterilizing immunity. Importantly, the enhanced protection observed with this strategy cannot be attributed solely to the route of administration, because its broader antigenic composition also targeted stages of pathogenesis preceding toxin-mediated injury. Both antigen breadth and immune compartmentalization may therefore contribute to the improved protection observed in this model.

A similar multivalent principle underlies the mRNA–lipid nanoparticle vaccine developed by Alameh et al.^189^, which incorporated antigens targeting toxins together with spore- and vegetative-cell-associated components. In preclinical models, this approach induced systemic and mucosal immune responses, reduced bacterial colonization, and protected against primary and recurrent CDI. These findings further support the concept that simultaneous targeting of toxin-mediated disease and pathogen colonization may provide broader protection than toxin-directed immunity alone. Likewise, engineered probiotic-based oral vaccines delivering toxin domains reduced bacterial burden, toxin expression, and intestinal injury while inducing both mucosal and systemic antibody responses in murine CDI models.^190^ Although promising, these approaches remain preclinical and require validation in humans.

Consistent with this shift toward broader protection, additional vaccine strategies have focused specifically on antigens involved in colonization and persistence. Several preclinical studies have identified conserved surface-associated antigens, including lipoproteins and cell wall proteins such as Cwp2, that induce humoral and mucosal immune responses and reduce bacterial adhesion, spore burden, and toxin levels in murine CDI models.^191^ ^,^ ^192^ These approaches are particularly relevant because they target stages of infection that precede toxin-mediated damage and may complement rather than replace antitoxin immunity. However, their translational potential remains to be established in human studies.

Emerging vaccine designs increasingly integrate these complementary principles. Multi-epitope mucosal platforms incorporating recombinant outer membrane vesicles and engineered *Bacillus subtilis* spores have been proposed to simultaneously target vegetative cells and spores while promoting systemic IgG and mucosal IgA responses.^193^ Similarly, oral chimeric constructs combining toxin antigens with colonization-associated factors aim to broaden immune coverage across multiple stages of CDI pathogenesis.^194^ Although these strategies are conceptually attractive because they may address colonization, persistence, and toxin-mediated disease simultaneously, their efficacy and safety remain to be validated experimentally and clinically.

Overall, clinical experience with *C. difficile* toxoid vaccines indicates that robust systemic antitoxin antibody responses alone are not a sufficient correlate of clinical protection. Whether this reflects inadequate toxin neutralization at the intestinal mucosa, the inability of toxin-directed immunity to control colonization and spore persistence, or a combination of these mechanisms remains unresolved. Emerging preclinical evidence supports broader vaccine strategies that combine toxin neutralization with mucosal immunity and antigenic targets associated with spores, vegetative cells, colonization, and persistence. However, the apparent advantages of these multivalent approaches currently derive predominantly from animal models, and their superiority over toxin-based vaccination remains to be demonstrated clinically. The principal active immunization strategies currently under investigation are summarized in Table 4.

**Table 4.** Overview of active immunization strategies against *C. difficile.*

Study/Strategy	Vaccine type	Target antigen(s)	Model	Key findings	References
Toxoid-based vaccines (Phase 2)	Toxoid (bivalent)	TcdA, TcdB	Humans	Strong and persistent neutralizing antibody responses; enhanced with higher doses and booster regimens	[181‐183]
Toxoid-based vaccines (Phase 3)	Toxoid (bivalent)	TcdA, TcdB	Humans	Failed to demonstrate efficacy in preventing primary CDI; trials terminated due to futility despite good safety	[184,185]
Early-phase clinical candidate	Protein+adjuvant	TcdA, TcdB	Humans	Safe and immunogenic, especially with adjuvant formulations	[180]
Maternal/passive immunity strategies	Recombinant toxin fragments	TcdA, TcdB	Animal models	Induced robust humoral responses and passive transfer of protective antibodies to offspring	[195]
Multivalent mucosal vaccine	Multicomponent mucosal vaccine	Toxin+spore+vegetative-cell-associated antigens	Mouse	Induced mucosal and tissue-resident immunity; enhanced pathogen clearance and protection against recurrence	[188]
mRNA-based vaccine platform	mRNA–LNP multivalent	Toxin, spore and vegetative-cell-associated antigens	Mouse	Induced systemic and mucosal immunity; protected against primary and recurrent infection; improved decolonization	[189]
Probiotic-based oral vaccine	Engineered live vectors	Toxin domains (e.g., tcdB)	Mouse	Reduced bacterial load and toxin expression; preserved intestinal integrity; induced systemic and mucosal responses	[190]
Surface antigen–targeted vaccines (lipoproteins)	Protein-based	LP1, LP2	Mouse	Induced humoral and cellular immunity; reduced adhesion, spore burden, and toxin levels	[191]
Surface antigen–targeted vaccines (cell wall proteins)	Protein-based	Cwp2	Mouse	Reduced colonization and toxin levels; inhibited bacterial adhesion; provided protection in CDI models	[192]
Mucosal multi-epitope strategies	OMVs+spores	Surface+spore antigens	Preclinical	Induced systemic IgG and mucosal IgA; potential to block colonization and transmission	[193]
Oral/mucosal chimeric constructs	Recombinant protein	TcdA/TcdB+colonization factors	Preclinical	Target both toxin-mediated damage and bacterial colonization; enhanced immune coverage	[194]

#### 3.3.3. Host-microbiota modulation

Host–microbiota interactions play a central role in both the pathogenesis and prevention of CDI. Antibiotic-induced dysbiosis disrupts colonization resistance, facilitating spore germination and vegetative outgrowth while reducing microbial diversity and beneficial taxa such as *Bacteroidetes*, *Prevotella*, and *Bifidobacterium.* ^196^ Dysbiosis in CDI is consistently characterized by reduced diversity, depletion of *Firmicutes* and *Bacteroidetes*, and expansion of opportunistic taxa such as *Proteobacteria* and *Enterobacteriaceae*, alterations that often precede treatment and become more pronounced in recurrent disease.^197^ ^,^ ^198^ Recent systems-level approaches further indicate that colonization resistance is an emergent property of microbial interaction networks rather than individual taxa, with computational models such as generalized microbe–phenotype triangulation demonstrating the predictive value of ecological community structure in determining susceptibility to *C. difficile* colonization.^199^

Mechanistically, CDI recurrence can be viewed as a sequential ecological process initiated by antibiotic-induced dysbiosis. Loss of microbial diversity and depletion of key commensal taxa impair colonization resistance, resulting in altered bile acid metabolism, reduced production of secondary bile acids and SCFAs, and increased availability of nutrients that support *C. difficile* germination and expansion.^200^ ^,^ ^201^ These changes facilitate spore germination, vegetative outgrowth, toxin production, and epithelial injury, which in turn promote inflammation and further disrupt microbial community structure. The resulting inflammatory and metabolic environment favors persistence of dysbiosis, delays recovery of protective microbial functions, and creates a self-reinforcing cycle that increases susceptibility to recurrent disease. Consequently, recurrence reflects not only pathogen persistence but also incomplete restoration of the ecological, metabolic, and immunological networks that normally maintain colonization resistance.^101^ ^,^ ^202^ ^,^ ^203^

Microbiota-derived metabolites are key regulators of this process. Bile acids represent major signaling molecules, with primary bile acids promoting spore germination and secondary bile acids inhibiting both germination and vegetative growth. These effects are influenced by microbial bile salt hydrolase activity and strain-specific metabolic capabilities, including those of *C. difficile* itself, which contribute to local bile acid remodeling and biofilm formation.^204‐206^ Clinical evidence further supports the importance of bile acid restoration in recurrence prevention. In a phase 3 trial, administration of the microbiome therapeutic SER-109 was associated with rapid microbial engraftment, recovery of bile acid profiles linked to inhibition of *C. difficile* spore germination, and a substantial reduction in recurrent CDI, supporting restoration of colonization resistance as a key therapeutic mechanism.^65^ Beyond bile acids, nutrient availability and metabolic competition strongly influence pathogen expansion. Increased availability of amino acids such as proline supports *C. difficile* growth, whereas commensal-mediated nutrient competition and niche occupation restrict pathogen proliferation.^200^ At the enzymatic level, Stickland fermentation pathways, particularly proline and glycine metabolism, are central to *C. difficile* energy production and virulence, with proline reductase emerging as a potential therapeutic target.^99^ ^,^ ^100^ ^,^ ^207^ These observations are consistent with experimental microbiome studies showing that proline utilization may be a stronger determinant of colonization success than bile acid alterations alone in certain antibiotic-disrupted communities.^99^ Recent studies further demonstrate that microbiota-derived metabolites, including aromatic amino acid derivatives such as 4-hydroxyphenylacetic acid, modulate epithelial and immune functions, highlighting the broader role of microbial metabolism in host–pathogen interactions.^208^

SCFAs, particularly butyrate, provide an important mechanistic link between microbiota composition, epithelial barrier integrity, and immune regulation. Reduced SCFAs concentrations are consistently associated with CDI, whereas restoration of SCFAs production correlates with recovery of microbiota function and improved discrimination between eubiotic and dysbiotic states.^202^ Consistent with these observations, recurrent CDI has been associated with depletion of SCFAs-producing taxa together with reduced concentrations of secondary bile acids, including lithocholic and deoxycholic acids, further linking metabolic dysfunction to recurrence risk.^209^ Longitudinal analyses further demonstrated that recovery of secondary bile acids and bile salt hydrolase activity occurs predominantly in patients who remain recurrence-free, supporting their role as functional markers of microbiome restoration and colonization resistance.^210^

The host immune status represents a critical determinant of colonization resistance. Intestinal inflammation alters microbial composition and promotes *C. difficile* persistence, while restoration of immune homeostasis facilitates recovery of a colonization-resistant ecosystem.^201^ These interactions extend to complex cross-kingdom effects involving commensal eukaryotes, fungal communities, and host immune signaling pathways, emphasizing the bidirectional nature of host–microbiota communication.^211^ Probiotic interventions further support this concept by exerting combined metabolic and immunomodulatory effects that enhance epithelial barrier function and reduce disease severity.^212^

**Figure 2.**
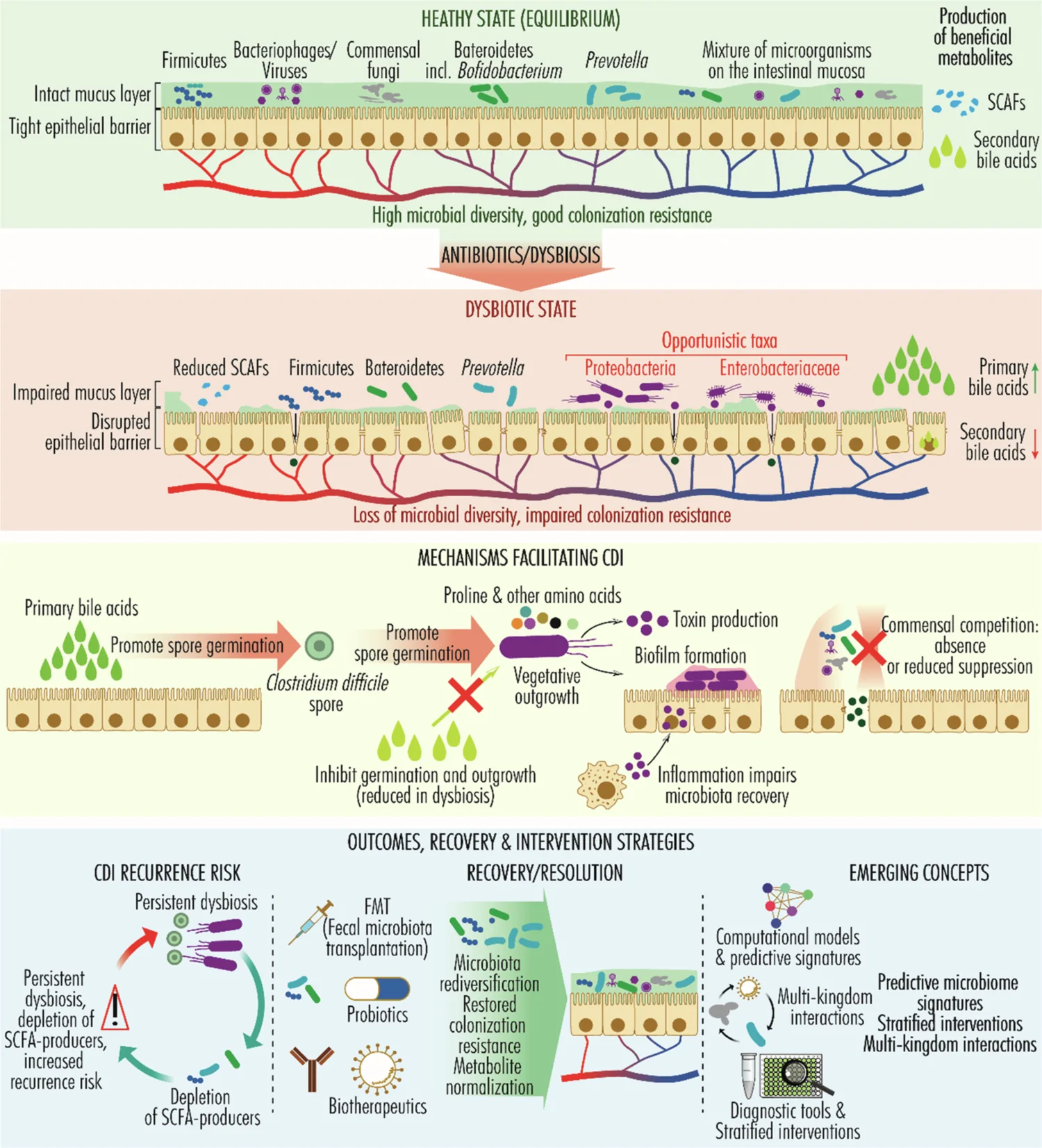
**Mechanistic interplay between microbiota composition, metabolic pathways, and host responses in CDI**. In the healthy state, a diverse gut microbiota maintains colonization resistance through intact epithelial barriers and the production of metabolites such as short-chain fatty acids (SCFAs) and secondary bile acids. Antibiotic-induced dysbiosis disrupts this equilibrium, reducing microbial diversity, impairing barrier function, and shifting bile acid metabolism toward primary bile acids that promote *C. difficile* spore germination. Loss of commensal competition and altered nutrient availability (e.g., amino acids) facilitate *C. difficile* outgrowth, toxin production, and biofilm formation, further amplifying inflammation and impairing microbiota recovery. Persistent dysbiosis increases recurrence risk, whereas restoration of microbial diversity and metabolic function (e.g., via FMT or microbiome-based therapeutics) supports recovery and re-establishes colonization resistance. Emerging approaches integrate multi-kingdom interactions, computational modeling, and microbiome-derived biomarkers to enable predictive and stratified therapeutic strategies (created with Adobe Illustrator CS6, Adobe Photoshop CS3).

These interconnected mechanisms linking microbial composition, metabolite dynamics, and host responses in CDI pathogenesis and recovery are summarized in Figure 2.

Clinical and longitudinal studies reinforce the importance of microbiota composition and function in determining disease outcomes. Restoration of microbial diversity is strongly associated with CDI resolution, whereas persistent dysbiosis correlates with recurrence risk. Therapeutic strategies such as fidaxomicin better preserve microbiome composition than vancomycin, while depletion of SCFA-producing taxa and alterations in bile acid metabolism show predictive value for recurrence, particularly in pediatric populations.^209^ ^,^ ^213^ Longitudinal metagenomic analyses further demonstrate dynamic shifts in microbial communities and metabolic pathways across colonization, infection, and recovery phases, linking reductions in SCFA-producing bacteria and alterations in fatty acid and bile acid metabolism to disease progression.^203^ ^,^ ^214^ Longitudinal multi-omics studies similarly showed that recurrent CDI is characterized by delayed recovery of microbial diversity, reduced microbial bile acid deconjugation activity, and persistent metabolic abnormalities, indicating that restoration of microbiome function is a critical determinant of sustained disease resolution.^215^ Microbiota recovery after CDI is often heterogeneous and incomplete, with persistent ecological instability observed despite probiotic supplementation or antibiotic withdrawal, underscoring the difficulty of fully restoring microbiome function.^216^ ^,^ ^217^ Emerging evidence also implicates fungal dysbiosis and alterations in the gut virome, including bacteriophage-mediated modulation of microbial networks and ecosystem resilience, in shaping recovery trajectories and recurrence risk.^218^ ^,^ ^219^

Importantly, microbiome-based stratification approaches are beginning to translate mechanistic insights into clinical applications. Recent studies demonstrate that microbiota composition can distinguish asymptomatic colonization from active CDI independently of diversity metrics, with specific taxa such as *Akkermansia muciniphila* and *Bifidobacterium adolescentis* associated with toxin-positive states, supporting the development of microbiome-informed diagnostic tools.^220^ In parallel, computational models integrating microbiome composition and metabolic outputs can predict individual susceptibility and guide the design of targeted interventions, including optimized probiotic consortia and next-generation live biotherapeutics.^101^

These findings indicate that CDI is not solely a toxin-mediated disease but a consequence of disrupted host–microbiota equilibrium involving interconnected ecological, metabolic, and immune mechanisms. Restoration of microbiota diversity and function through FMT and other microbiome-directed interventions therefore represents a central strategy for re-establishing colonization resistance, reducing recurrence risk, and promoting durable recovery.

## 4. Challenges and limitations

Early microbiome research was limited by small datasets, technical constraints, and confounding environmental factors, delaying causal understanding and clinical translation; however, recent large-scale multi-omics datasets and computational advances have begun to establish robust links between microbiome composition, function, and human health, enabling the development of microbiome-targeted therapeutics.^221^

**Figure 3.**
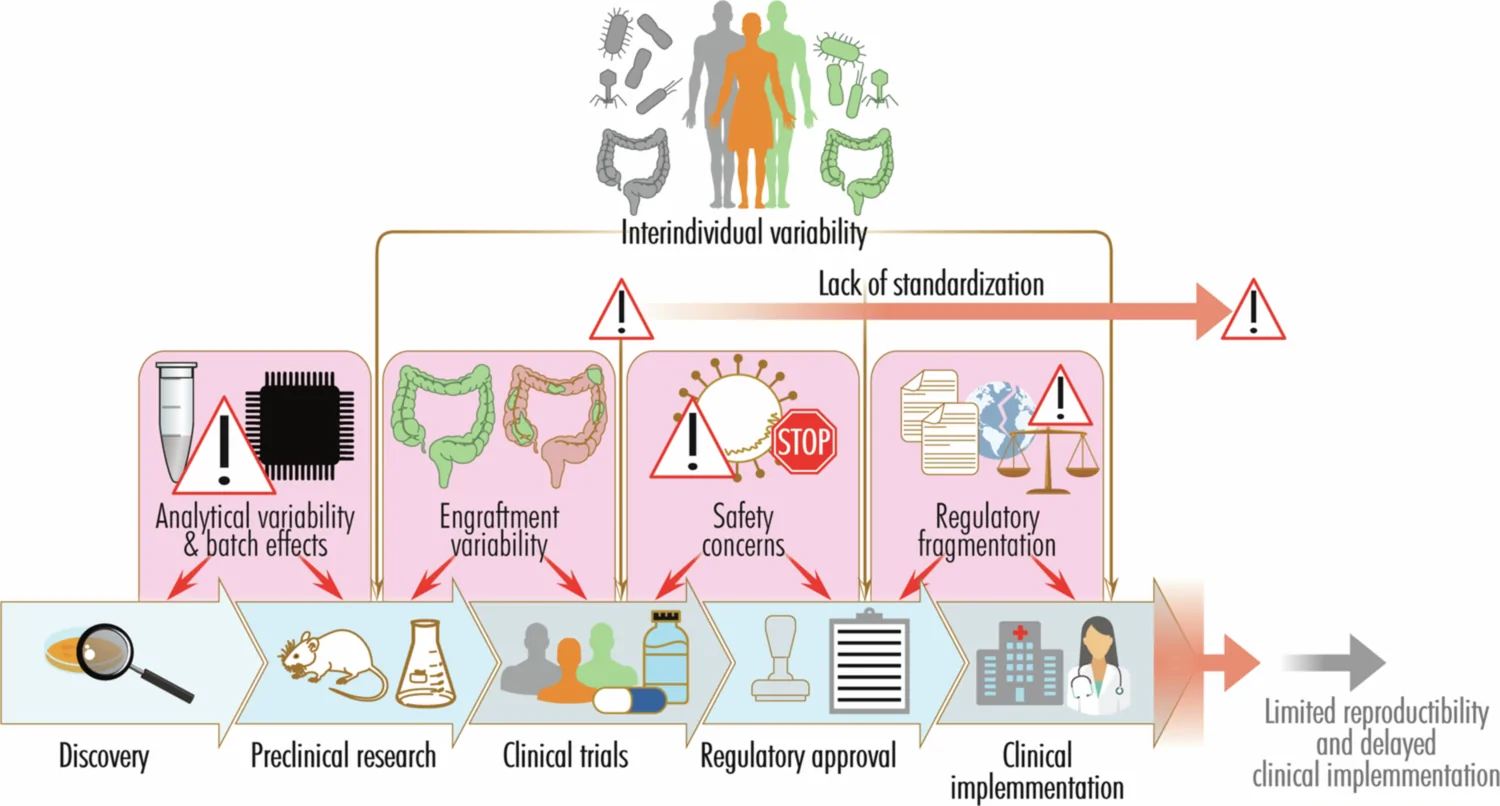
**Translational barriers limiting the clinical implementation of microbiome-based therapeutics.** The translation of microbiome-based therapeutics from discovery to clinical practice is constrained by multiple interconnected barriers. Analytical variability and batch effects affect early-stage characterization, while interindividual variability and inconsistent microbial engraftment limit predictability in preclinical and clinical settings. Safety concerns and fragmented regulatory frameworks further complicate clinical development and approval. A lack of standardization across sampling, manufacturing, and analytical pipelines amplifies these challenges throughout the translational pathway. These factors contribute to limited reproducibility, reduced comparability across studies, and delayed clinical implementation (created with Adobe Illustrator CS6, Adobe Photoshop CS3).

Despite significant progress, the translation of microbiome-based therapeutics into routine clinical practice faces several important challenges. A major limitation is the scarcity of large-scale, randomized, multicenter clinical trials. While numerous pilot studies and early-phase trials have demonstrated promising safety and efficacy, the absence of standardized study designs and long-term follow-up makes it difficult to draw definitive conclusions about their reproducibility and therapeutic potential. These key translational and methodological barriers are summarized in Figure 3.

Another critical barrier is the high degree of interindividual variability in the human microbiome. Each individual harbors a unique microbial signature shaped by genetics, diet, lifestyle, and prior antibiotic exposure, making therapeutic responses difficult to predict and generalize. Recent clinical and metagenomic studies demonstrate that variability in treatment outcomes is closely linked to donor–recipient compatibility and microbial engraftment dynamics.

Successful engraftment of donor-derived strains strongly correlates with clinical response, with responders showing rapid microbiota restructuring and sustained persistence of donor taxa.^222^ ^,^ ^223^ In contrast, incomplete or unstable engraftment is associated with therapeutic failure, highlighting the importance of ecological compatibility, niche availability, and baseline microbiome composition as key determinants of treatment success.^224^ ^,^ ^225^ However, engraftment remains difficult to quantify accurately, as current metagenomic approaches are limited by strain-level resolution and susceptibility to analytical artifacts, complicating prediction and interpretation of therapeutic outcomes.^45^ ^,^ ^46^

A major but often underappreciated limitation in microbiome research is the presence of batch effects arising from differences in sample processing, sequencing, and data handling, which can introduce spurious associations and obscure true biological signals. Conventional correction methods, largely adapted from transcriptomics, often fail to account for the specific characteristics of microbiome data, such as zero inflation and overdispersion, highlighting the need for dedicated statistical frameworks.^226^ ^,^ ^227^

Safety concerns also remain a pressing issue. Although generally well tolerated, interventions such as FMT carry a potential risk of transmitting opportunistic pathogens, including antibiotic-resistant strains. A sentinel example was reported by DeFilipp et al.^228^, who documented transmission of an extended-spectrum *β*-lactamase (ESBL)-producing *Escherichia coli* through donor-derived FMT material, resulting in invasive infections in two recipients, one of whom died. Importantly, the implicated material had been manufactured before donor screening for ESBL-producing organisms was incorporated into screening protocols and was subsequently administered from stored preparations. This case therefore illustrates not only the potential infectious risks of FMT, but also the critical importance of updated donor screening, appropriate management of previously manufactured material, and rigorous biobanking and quality-control procedures.

Regulatory and standardization challenges further complicate clinical translation. Currently, there is no globally unified framework for the classification, quality control, and approval of microbiome-based therapeutics. In the United States, FMT and LBPs are regulated as biologics, whereas in Europe their legal status varies across countries, complicating multinational clinical development. Recent approvals of FDA-regulated microbiome therapeutics, including Rebyota™ (2022) and Vowst™ (2023), represent important milestones, yet also highlight the need for harmonized international guidelines and standardized manufacturing processes.^25^

A key limitation in microbiome-based therapeutics is the lack of standardized potency and quality control frameworks for LBPs. Conventional CFU-based assays often fail to capture functional activity, particularly in multi-strain formulations, while alternative methods remain technically complex and insufficiently standardized.^229^ In parallel, existing regulatory frameworks for microbiological safety lack the specificity required to distinguish contaminants from product strains, underscoring a critical gap between product characterization and clinical efficacy.^25^ Although recent initiatives, such as the EU SoHO regulation, aim to expand and harmonize regulatory oversight of human microbiome-derived materials, variability in sampling, processing, and analytical pipelines continues to limit reproducibility and comparability across studies.^230^ ^,^ ^231^

Furthermore, real-world clinical data indicate that therapeutic efficacy is tightly linked to microbiota restructuring and metabolic recovery. For example, treatment with fecal microbiota, live-jslm (REBYOTA™) is associated with coordinated restoration of microbial composition and bile acid metabolism in responders, whereas non-responders exhibit incomplete or unstable microbiome recovery, reinforcing the importance of functional ecosystem restoration as a determinant of clinical success.^232^

Although microbiome-derived biomarkers show promising predictive value across multiple diseases, their clinical implementation remains limited by methodological heterogeneity, inconsistent associations across cohorts, and lack of robust external validation.^233^ ^,^ ^234^ Emerging evidence suggests that microbiome signatures may outperform conventional clinical markers in predicting treatment response, further highlighting their potential while underscoring the need for standardized validation frameworks. Notwithstanding advances in sequencing technologies and multi-omics approaches, the qualification and clinical implementation of microbiome-based biomarkers remain hindered by fragmented standardization, inconsistent metadata reporting, and substantial interindividual variability.^24^

These challenges underscore a fundamental translational gap in microbiome research, where rapid technological and biological advances continue to outpace the development of standardized, reproducible, and clinically actionable frameworks required for reliable implementation in precision medicine.

## 5. Perspectives

Research in CDI is increasingly exploring precision microbiome approaches in which microbial, metabolic, and host-derived biomarkers could complement established clinical factors for risk stratification and therapeutic decision-making. The integration of metagenomics, metabolomics, transcriptomics, proteomics, and advanced computational approaches is improving our understanding of host–microbiome interactions and providing new opportunities for patient stratification, recurrence prediction, and treatment selection.^235^ ^,^ ^236^

A growing body of evidence suggests that functional ecosystem signatures may provide greater clinical value than taxonomic profiles alone. Prospective microbiome studies have shown that patients who subsequently develop CDI exhibit distinct microbial signatures before disease onset, supporting the development of microbiome-based risk stratification tools.^197^ Similarly, metabolomic investigations have identified alterations in bile acid metabolism, short-chain fatty acids, amino acid pathways, and other microbial metabolites associated with CDI diagnosis and recurrence risk.^215^ ^,^ ^237^ ^,^ ^238^ Recent studies integrating microbiome composition, metabolomics, immune markers, and clinical variables have further demonstrated the potential to identify patients at increased risk of recurrence and guide early use of targeted interventions such as fidaxomicin, bezlotoxumab, or FMT.^239‐241^ The BEYOND study further highlighted the value of integrated host–microbiome biomarkers by combining microbial signatures, inflammatory markers, host genetic factors, and clinical parameters into a predictive score capable of identifying patients at high risk of relapse, organ dysfunction, or death.^167^

Artificial intelligence and machine learning are emerging as important tools for translating these multidimensional datasets into clinically actionable models. AI-driven approaches have shown potential for predicting CDI recurrence, identifying individuals at increased risk before disease onset, and supporting antimicrobial stewardship strategies.^242‐244^ However, real-world implementation studies indicate that predictive performance alone does not guarantee improved clinical outcomes, emphasizing the need for effective integration into clinical workflows, external validation, model interpretability, and standardized analytical pipelines.^245^

Beyond risk prediction, computational and ecological modeling are creating opportunities for the rational design of microbiome therapeutics. Machine-learning–integrated ecological models and genome-scale metabolic frameworks can identify microbial configurations associated with colonization resistance and predict responses to microbiome interventions.^246‐248^ These approaches are increasingly being applied to the development of defined microbial consortia and next-generation live biotherapeutic products. Recent studies have identified protective bacterial strains associated with successful FMT outcomes, developed computational frameworks for consortium selection, and demonstrated that donor–recipient microbiome compatibility may influence therapeutic efficacy^101^ ^,^ ^249-251^. Together, these findings provide a foundation for the rational design of microbiome therapeutics and, potentially, more individualized intervention strategies.

Microbiome engineering and synthetic biology may further expand these possibilities through programmable bacterial strains capable of delivering therapeutic metabolites, neutralizing toxins, or modulating host immunity.^252‐255^

Synthetic biology and niche-engineering strategies may also help overcome the colonization resistance that limits engraftment of exogenous therapeutic strains. Experimental studies have shown that providing introduced gut bacteria with access to otherwise underutilized dietary substrates can create exclusive or orthogonal metabolic niches that facilitate their establishment within an intact microbiota.^256^ ^,^ ^257^ Building on this concept, a genetically engineered *Phocaeicola vulgatus* strain combining porphyran-dependent reversible engraftment with an engineered oxalate-degradation pathway achieved controllable colonization in humans in a phase 1/2a trial.^258^ Although these approaches have not yet been established for CDI, they provide proof of concept that metabolic niche engineering can improve the engraftment and controllability of therapeutic bacteria within established gut microbial communities.

At the same time, systems biology approaches, digital twin technologies, and multi-omics integration may provide increasingly detailed representations of host–microbiome dynamics, although substantial challenges related to validation, scalability, standardization, and regulatory acceptance remain.^259‐261^

Despite this potential, a substantial gap remains between discovery-oriented precision microbiome research and routine CDI care. Repeated metagenomic, metabolomic, or multi-omics profiling may be informative in research settings and clinical trials, but methodological complexity, lack of standardization, infrastructure requirements, and challenges related to integration into clinical workflows currently limit their routine implementation.^32^ ^,^ ^262^ For the foreseeable future, antibiotics are likely to remain the foundation of CDI treatment, with established adjunctive and microbiota-based interventions used according to recurrence risk, eligibility, availability, and local resources.^263^ The near-term translational value of multi-omics and computational studies may lie less in implementing comprehensive longitudinal profiling for individual patients than in identifying robust biomarkers that can ultimately be converted into simpler and clinically deployable assays.^262^ ^,^ ^264^ Accordingly, widespread precision-guided CDI management will depend not only on improved biological understanding and predictive performance, but also on the development of scalable and affordable diagnostic tools with clinically actionable turnaround times that can be integrated into routine laboratory workflows.

Successful clinical translation will also require standardized manufacturing processes, rigorous safety assessment, harmonized regulatory frameworks, reproducible analytical pipelines, and evidence that precision-guided approaches provide clinically meaningful benefit beyond existing treatment algorithms.^24^ Thus, multi-omics, artificial intelligence, and ecological modeling should currently be viewed primarily as discovery and stratification tools rather than routine components of CDI management. Their ultimate clinical value will depend on whether complex biological signatures can be translated into validated, accessible, and cost-effective diagnostic or therapeutic decision tools. A conceptual roadmap integrating these developments, while distinguishing discovery-oriented approaches from potential clinical applications, is summarized in Table 5.

**Table 5.** Conceptual roadmap toward precision microbiome therapeutics in CDI.

Stage	Core concept	Key approaches	Biological focus	Clinical output
Multi-omics integration	High-resolution characterization of the microbiome and host interactions	Metagenomics, metabolomics, transcriptomics, proteomics	Microbial composition, metabolic pathways, host responses	Identification of biomarkers for CDI diagnosis and recurrence prediction
AI & machine learning	Integration and interpretation of complex datasets	Predictive modeling, biomarker discovery, response prediction	Host–microbiome interaction patterns, treatment response	Risk stratification and prediction of recurrent CDI and treatment response
Microbiome engineering	Design of targeted and programmable interventions	Engineered probiotics, live biotherapeutics, synthetic consortia	Metabolite production, toxin neutralization, immune modulation	Rational design of live biotherapeutics and microbial consortia for CDI
Systems biology & ecological modeling	Mechanistic understanding of microbiome dynamics	Network analysis, metabolic modeling, ecological simulations	Microbial interactions, metabolic networks, ecosystem resilience	Prediction of colonization resistance and optimization of microbiome interventions
Clinical translation	Translation of validated biomarkers into scalable decision-support tools	Simplified biomarker assays, validated risk models, point-of-care diagnostics	Clinically actionable markers of recurrence and treatment response	Risk-guided treatment selection using accessible and validated clinical tools

Despite persistent challenges related to data standardization, model interpretability, external validation, and interindividual variability, the integration of multi-omics profiling, ecological modeling, artificial intelligence, and rational microbiome engineering is creating a pathway toward precision CDI management. Rather than relying solely on standardized treatment algorithms, future therapeutic strategies may incorporate patient-specific microbial, metabolic, and immune signatures to guide recurrence risk assessment, treatment selection, and restoration of colonization resistance. These advances position CDI as a leading model for the clinical translation of precision microbiome medicine.

## 6. Conclusions

CDI represents one of the clearest examples of a dysbiosis-driven disease, in which disruption of microbial ecology, loss of colonization resistance, and metabolic imbalance contribute directly to pathogen expansion, toxin production, and recurrence. Consequently, CDI has emerged as a leading model for the development and clinical evaluation of microbiome-based therapeutics.

The evidence reviewed here indicates that successful CDI management extends beyond pathogen eradication and increasingly depends on restoration of microbiome function. Additive approaches, including fecal microbiota transplantation, live biotherapeutic products, and defined microbial consortia, aim to re-establish microbial diversity and colonization resistance. Subtractive strategies, such as microbiome-sparing antibiotics, bacteriocins, bacteriophages, and CRISPR-based antimicrobials, seek to selectively eliminate *C. difficile* while minimizing collateral ecological damage. Modulatory interventions, including bile acid manipulation, anti-toxin therapies, immunological approaches, and metabolite-directed strategies, further highlight the importance of targeting the biological processes that sustain disease persistence and recurrence.

Despite substantial progress, important challenges remain, including interindividual variability, incomplete understanding of engraftment dynamics, limited predictive biomarkers, and the need for standardized manufacturing and regulatory frameworks. Future advances will likely depend on integrating microbial, metabolic, and host-derived biomarkers with rationally designed microbiome therapeutics to support recurrence prediction, patient stratification, and personalized treatment selection.

Together, these developments support a transition from pathogen-centered treatment toward ecological restoration as a therapeutic objective. As microbiome science continues to mature, CDI is likely to remain a key model for the translation of precision microbiome medicine into clinical practice.

## Data Availability

Data sharing is not applicable to this article as no datasets were generated or analysed during the current study.
